# Effects and Mechanism of Huannao Yicong Decoction Extract on the Ethology of Transgenic APP/PS1 Mice

**DOI:** 10.1155/2017/9502067

**Published:** 2017-12-20

**Authors:** Meixia Liu, Yun Wei, Yang Yang, Longtao Liu, Lin Liang, Jiangang Liu, Hao Li

**Affiliations:** ^1^Xiyuan Hospital, China Academy of Chinese Medical Sciences, Beijing 100091, China; ^2^Beijing Hospital of Traditional Chinese Medicine, Beijing 100010, China

## Abstract

To investigate the mechanism of Huannao Yicong Decoction (HYD) extract on improving of learning memory of transgenic amyloid precursor protein (APP)/presenilin 1 (PS1) mice, we randomly divided 60 transgenic APP/PS1 mice of 3 months old into 4 groups: the model group, the Donepezil group, the HYD-L group, and the HYD-H group, with 15 C57BL/6J mice of the same genetic background as the control group. These mice were gavaged for 6 months in a row. The results showed that the latency was significantly shortened and the number of passing through the original platform was increased. HYD extract can increase the amount of neurons and improve the morphological structure of Nissl body obviously. The *γ*-secretase activity and the expression of phosphorylated APP, A*β*1-40, and A*β*1-42 in hippocampal CA1 were significantly decreased. The expressions of protein and mRNA of PEN-2 and CREB in hippocampal were significantly downregulated. These results demonstrated that HYD extract can improve the memory ability of transgenic APP/PS1 mice, which was related to the protection of neurons and structure of Nissl body, reducing cleavage of APP and production of A*β* and inhibiting the activity of *γ*-secretase by decreasing CREB activity because of downregulated expression of PEN-2.

## 1. Introduction

The aging of population has become an irreversible trend in 21st Century. With memory and cognitive impairment as the main manifestation of Alzheimer's disease (AD) and its incidence rising, it is a serious threat to the health and life quality of elderly people. Composing of Radix polygonum multiflorum preparata,* Panax ginseng*,* Acorus gramineus*,* Coptis chinensis*, and* Ligusticum wallichii*, Huannao Yicong Decoction (HYD) is a clinical protocol, which is used to treat the elderly patients with cognitive impairment and Alzheimer's disease in Xiyuan Hospital of China Academy of Chinese Medical Sciences and has obtained the national invention patent [1] and has been applied in clinic for more than 10 years. The previous clinical studies had confirmed its effect on improving memory and cognition [2]. Basic research showed that HYD can improve the behavior of AD model rats, pathological morphology, and cholinergic system, inhibit the apoptosis of nerve cells, regulate the protein expression related to APP and A*β*, and inhibit inflammatory factors and have the effect of antioxidation [3]. This study is mainly to investigate the mechanism of HYD extract on improving the learning memory of transgenic amyloid precursor protein (APP)/presenilin 1 (PS1) mice as animal model simulating AD through the detection of ethology, pathological morphology, and related indicators of *γ*-secretase.

## 2. Materials and Methods

### 2.1. Animals

60 transgenic APP/PS1 mice and 15 C57BL/6J mice of a congenic genetic background, 3 months old, clean grade (SPF grade), male and female combination, weight (25–35) g, were obtained from Chinese Academy of Medical Sciences (license number: SCXK (Beijing) 2009-0007). The experiment was carried out after 7 days of adaptive feeding in the room where the temperature was controlled at 23°C~25°C and the humidity was 50%~70%.

### 2.2. Drugs and Reagents

Donepezil hydrochloride (Aricept, 5 mg/tablet, batch number: 100806A) was produced by Eisai Pharmaceutical Co. Ltd. (China). HYD extract (3.2 g crude drug/g) was prepared in the drug preparation room, Beijing University of Chinese Medicine. Chinese herbal pieces, including Radix polygonum multiflorum preparata,* Panax ginseng*,* Acorus gramineus*,* Coptis chinensis*, and* Ligusticum wallichii*, were obtained from Hebei Shennong Pharmaceutical Co. Ltd. (Beijing). Rabbit anti-phosphorylation APP/ABPP (Thr668) (batch number: 990182w), Rabbit anti-A*β*1-40 (CT) (batch number: 20120810), Rabbit anti-A*β*1-42 (batch number: YE0907), Immunohistochemical staining kit (batch number: 999098), and DAB staining kit (batch number: 980579) were purchased from Beijing Bioss Technology Co. Ltd. Rabbit anti-anterior pharynx defective protein-1 (APH-1) (model: AB9214, Millipore), Rabbit anti-hypoxia inducing factor-1a (HIF-1a) (model: ab51608, Abcam), Rabbit anti-presenilin enhancer-2 (PEN-2) (model: SC-32946, Santa), Rabbit anti-cyclic adenosine monophosphate response element binding protein (CREB) (model: 06-863, Millipore), Mouse anti-*β*-actin antibody (model: TDY041, TiandeYue), Goat anti-rabbit IgG (H + L) (model: s004, FanxingBoao), and Luciferase assay kits (product code: E1910) were purchased from Promega company. Ultrapure RNA extraction kit (product code: CW0581), HiFi-MMLV cDNA first chain synthesis kit (product code: CW0744), Real Super Mixture (with Rox) (product code: CW0767), and DNase 1 (product code: CW2090) were purchased from Beijing Kangwei century biological technology Co. Ltd.

### 2.3. HYD Extract Preparation

**Figure 1:**
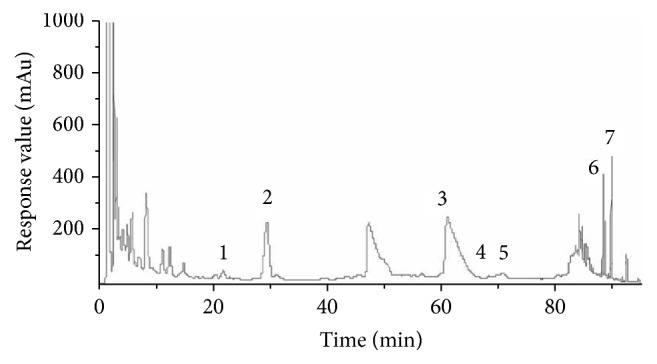
Fingerprint map of HYD extract detected with HPLC. (1) Ferulic acid; (2) styrene glycosides; (3) berberine hydrochloride; (4) ginseng saponin Rg1; (5) ginseng saponin Rb1; (6) ginseng saponin Re; (7) emodin.

The first step is to extract the volatile oil from* Acorus gramineus*, after which the drug liquid and residue together with Radix polygonum multiflorum preparata were extracted with water and precipitated with alcohol, and then the alcohol deposit liquid was obtained.* Panax ginseng*,* Coptis chinensis,* and* Ligusticum wallichii* were extracted together with alcohol to get the alcohol extract. The alcohol deposit liquid and alcohol extract were rotary evaporated together and then were mixed with the volatile oil, form which the final HYD extract was prepared. The HYD extract was detected by high performance liquid chromatography (HPLC) according the best chromatographic conditions which were the wavelength of 203 nm and 280 nm, mobile phase of acetonitrile - 0.05% phosphoric acid water, Apollo C18 (150 mm × 4.6 mm), column temperature 30°C, and the flow rate of 1.0 ml/min. The main effective components of HYD extract include ferulic acid, stilbene glycoside, berberine hydrochloride, ginseng saponin Rg1, Rb1, and Re, and emodin whose concentrations were 2.4183, 0.8765, 1.0424, 0.3993, 0.5816, 0.6932, and 0.8045 mg/g, respectively, reaching the requirements of Chinese Pharmacopoeia (2010 version). The quality was controlled with the combination of qualitative and quantitative methods, and the corresponding fingerprint was established (Figure 1).

### 2.4. Animal Grouping and Treatments

60 transgenic APP/PS1 mice were randomly (applying SPSS software) divided into 4 groups with 15 in each: model group (given the equal volume of distilled water), Donepezil group (given the Donepezil 0.65 mg/kg/d which is clinical equivalent dose), the HYD-L group (1.7 g/kg/d which is clinical equivalent dose), and the HYD-H group (3.4 g/kg/d which is twice the clinical equivalent dose), with 15 of C57BL/6J mice as control group (given the equal volume of distilled water). The mice of the five groups were gavaged for 6 months in a row, with the liquids of the equal volume and corresponding concentration diluted by distilled water. There were 6 mice dead for the gavage reason during the experiment, 2 in the Donepezil group, 1 in HYD-L group, and 3 in HYD-H group. The experiment was in accordance with the animal ethics requirements.

### 2.5. Morris Water Maze Test

The Morris water maze test was carried out after 6-month gavage, to assess the learning memory ability of the transgenic APP/PS1 mice. The Morris water maze apparatus (120 cm in diameter and 50 cm in depth) was produced by Institute of Pharmacology, Chinese Academy of Chinese Medicine. The water in the pool was 30 cm deep and approximately 1 cm above the platform surface, which was located in the fourth quadrant, with the temperature of 25 ± 1°C. Some milk powder was added in the water to make it opaque white, making the mice (black) unable to distinguish the platform in the tank. The whole experiment was divided into two parts: place navigation test and spatial probe test. For the place navigation test, at the marked entry point from the second quadrant 1/2 radian of the pool wall, the mice were put into the water against the pool wall one by one. Record the time (name as escape latency) the mice spend finding the platform (until the limbs on the platform) and the swimming distance by an autotracking system. Animals were allowed to swim for 3 min to search for the platform at each trial and to take a rest on platform for 10 s if they found it. For those animals who failed the mission, the escape latency was recorded as 180 s. All the mice were trained once a day and for 4 consecutive days. On the fifth day, the escape latency as an evaluation index of the learning memory ability of mice was recorded in the morning. In the afternoon the spatial probe test was performed to assess the spatial memory ability with removal of the platform. Choose an entry point, put the mice into the water with mice facing the pool wall, and record the times of crossing the platform-site in 3 min.

All mice were sacrificed by decapitation after the Morris water maze test. The brain was removed immediately after the transcardial perfusion with saline. The brains were sagittally cut into brain specimens of left or right sides allocated randomly on the ice. Six specimens in each group were fixed in 4% neutral paraformaldehyde solution and were sliced after paraffin embedded, which were used to observe after Nissl Staining and immunohistochemistry. The hippocampi of other specimens were isolated and stored in refrigerator at −80°C, in order to detect the related molecules by dual-luciferase reporter, Western blotting, and real-time fluorescent quantitative-polymerase chain reaction (RT-PCR).

### 2.6. Nissl Staining

The paraffin-embedded brain slices were deparaffinized and washed routinely, washed 1-2 min with distilled water, dipped for 30 min in 1% thionin water solution at 37°C incubator, then washed 1-2 min with distilled water, differentiated to see clear Nissl body with 95% alcohol, dehydrated for 8–10 min with anhydrous alcohol, permeabilized for 8–10 min with xylene, and sealed with neutral gum. Neurons and Nissl bodies in hippocampus CA1 area were observed by BH-2 biological microscope (produced by Olympus of Japan).

### 2.7. Immunohistochemistry

All paraffin-embedded brain slices were deparaffinized routinely and washed with distilled water, dipped in PBS for 5 min, incubated for 20 min in 3% H_2_O_2_ deionized water to eliminate the endogenous peroxidase activity, and incubated for 20 min at room temperature after working solution of normal goat serum was dropped for sealing; then the liquid was poured out with no washing. Then the slices were incubated overnight at 4°C with primary antibodies, which were diluted by appropriate proportion. On the second day, the slices were incubated at room temperature for 20 min with the secondary antibodies, before and after which the slices were washed 3 times with PBS (each for 3 min). After incubation at room temperature for 20 min with the working solution of horseradish peroxidase labeled streptavidin, the slices were washed 3 times with PBS, each for 3 min, stained with DAB, and then washed fully with tap water, redyed, dehydrated, transparentized, and sealed finally. The hippocampus CA1 area of mice was observed with BH-2 biological microscope. The positive result was brown yellow cytoplasm or prominence. Two slices from each mouse were observed and taken pictures of, applying the Image Pro Plus 6.0 image analysis software (produced by American Media Cybernetics corporation) to calculate the average optical density (OD) value of positive expression in each high power (×600) view.

### 2.8. Dual-Luciferase Reporter

Five hippocampus tissue specimens in each group were chosen and grinded with 200 ul lysates of luciferase assay kits, centrifuged for 5 min with the speed of 15000*g*. Then 20 ul of the supernatant was sucked out, which was blended with 100 ul Luciferase Assay Buffer II, The luciferase value was read using multifunctional microplate reader (produced by Molecular Devices corporation). Then 100 ul of stop&Glo Buffer was added and mixed well; then the* Renilla* value was recorded. Luciferase/*Renilla* value indicated *γ*-secretase activity with* Renilla* value as internal reference.

### 2.9. Western Blotting

Six samples in each group were used to extract the tissue proteins and protein quantifying by BCA kit. According to the loading quantity of sample of sodium dodecyl sulfate polyacrylamide gel electrophoresis (SDS-PAGE), the protein concentrations were adjusted with RIPA and 5x buffer solution of restored sample and the protein solution were boiled for 5 min. According to the molecular weights of target proteins, separation gels of 8% and 12% were prepared, respectively; the concentration of spacer gel was made at 5%. For electrophoresis, spacer gel constant voltage was set at 90 v for about 20 min. Separation gel constant voltage was set at 160 v, and the stop time of the electrophoresis was determined by predyed marker protein. Then the proteins were transferred onto the membranes, sealed, and incubated with primary antibodies and the membranes were washed. The proteins were incubated with the internal reference, which was *β*eta-actin mouse monoclonal antibody diluted with 3% BSA–TBST (rate 1 : 5000), for 2 h at room temperature. After incubating with the secondary antibody, the membranes were washed again. After the ECL reaction, the film was exposed, developed, and fixed.

### 2.10. RT-PCR

Six samples in each group were selected and the total RNA were extracted with ultrapure RNA extraction kit. Electrophoresis was performed on 5 ul of the RNA liquid with 1% agarose gel with DYY-GC electrophoresis apparatus (produced by Beijing Liuyi Instrument Factory). Primers were synthesized by Beijing Huateng Shengwei Biological Technology Co. Ltd., with *β*-actin as internal reference. The upstream of APH-1a mRNA primer was 5′-ATGCACCTTCGTCGCGTTCG-3′ and the downstream was 5′-AGAAGGACAGAGACAGCAGCACCAA-3′ (amplified length was 222 bp). The upstream of HIF-1*α* mRNA primer was 5′-GATGAATCAAAAACAGAGACGAAGG-3′ and the downstream was 5′-CTGATGCCTTAGCAGTGGTCGT-3′ (amplified length was 109 bp). The upstream of PEN-2 mRNA primer was 5′-ACACAGAGCAGAGCCAAATCAAAGG-3′ and the downstream was 5′-CAGGGGAATGGTGAAGGAGAGGTAG-3′ (amplified length was 155 bp). The upstream of CREB mRNA primer was 5′-ACCATTGCCCCTGGAGTTGTTAT-3′ and the downstream was 5′-CTCTTGCTGCCTCCCTGTTCTTC-3′ (amplified length was 115 bp). The upstream of internal reference *β*-actin mRNA primer was 5′-GCCTTCCTTCTTGGGTAT-3′ and the downstream was 5′-GGCATAGAGGTCTTTACGG-3′ (amplified length was 97 bp). Reverse transcription was conducted using the first strand synthesis kit of HiFi-MMLV cDNA and the amplification was conducted with RealSuper Mixture (with Rox) with the procedure of 95°C for 10 min, 40 cycles with melting for 15 s at 95°C and annealing and extension for 1 min at 60°C. The melting curve was analyzed at 60–95°C, after which the standard curve of the target gene and internal reference gene was drawn. At last the data was relatively quantified and analyzed using the method of 2^−ΔΔCT^.

### 2.11. Statistical Analyses

The data were expressed as the mean ± SEM. All statistical analyses were done by using the statistical package of SPSS 17.0. One-way ANOVA and *q*-test were used to compare among groups after all the data were tested with the normality test and homogeneity test of variance. *P* values of less than 0.05 were considered significant and those less than 0.01 were considered obviously significant.

## 3. Results

### 3.1. Effects of HYD Extract on the Learning Memory Ability of Transgenic APP/PS1 Mice

**Figure 2:**
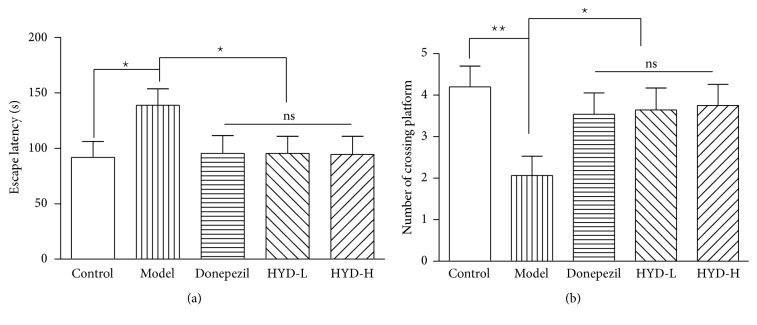
HYD extract improved the ability of learning and memory of transgenic APP/PS1 mice. (a) Escape latency, (b) number of crossing platform. ^★^*P* < 0.05, ^★★^*P* < 0.01.

As shown in Figure 2, compared with the control group, the escape latency of model group mice was significantly prolonged (*P* < 0.05); the number of crossing platform was decreased significantly (*P* < 0.01); after intervention, compared with the model group, the latencies of Donepezil group, HYD-L group, and HYD-H group were shortened (*P* < 0.05); the number of crossing platform was increased (*P* < 0.05) (Figure 2).

### 3.2. Effects of HYD Extract on Neurons and Nissl Body of Hippocampal CA1 Area of Transgenic APP/PS1 Mice

**Figure 3:**
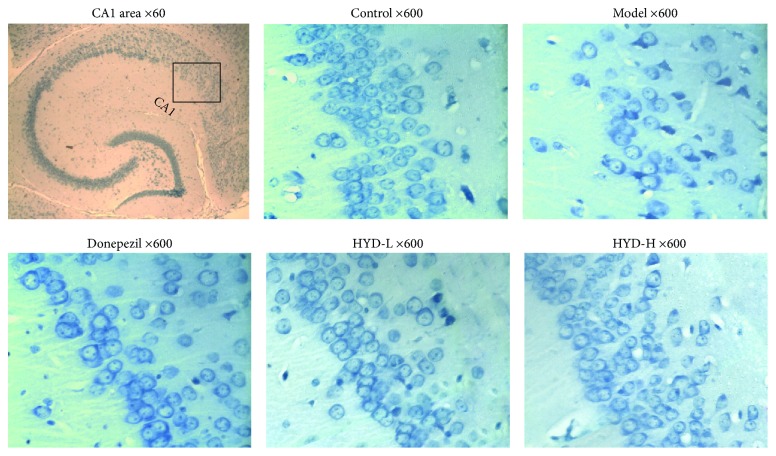
HYD extract protected the neuron and Nissl body of transgenic APP/PS1 mice (Nissl Staining, ×60 and ×600).

As shown in Figure 3, with Nissl Staining, the neurons of control group mice in hippocampus CA1 area were in a larger quantity, arranged in neat rows, rich in Nissl bodies (dark blue), with pale blue nucleus and light blue background; the neurons of model group mice in the hippocampal CA1 area decreased in quantity, arranged disorderedly, with obscure or disappeared boundary of Nissl bodies, karyopyknosis, or karyolysis which were shaped in elliptic or triangle and dyed dark blue; the neurons of Donepezil group, HYD-L group, and HYD-H group mice in the hippocampal CA1 area increased significantly in quantity, arranged neatly, with more Nissl bodies, little karyopyknosis, and karyolysis (Figure 3).

### 3.3. Effects of HYD Extract on the Expression of APP and A*β* in Hippocampal CA1 Area of Transgenic APP/PS1 Mice

**Figure 4:**
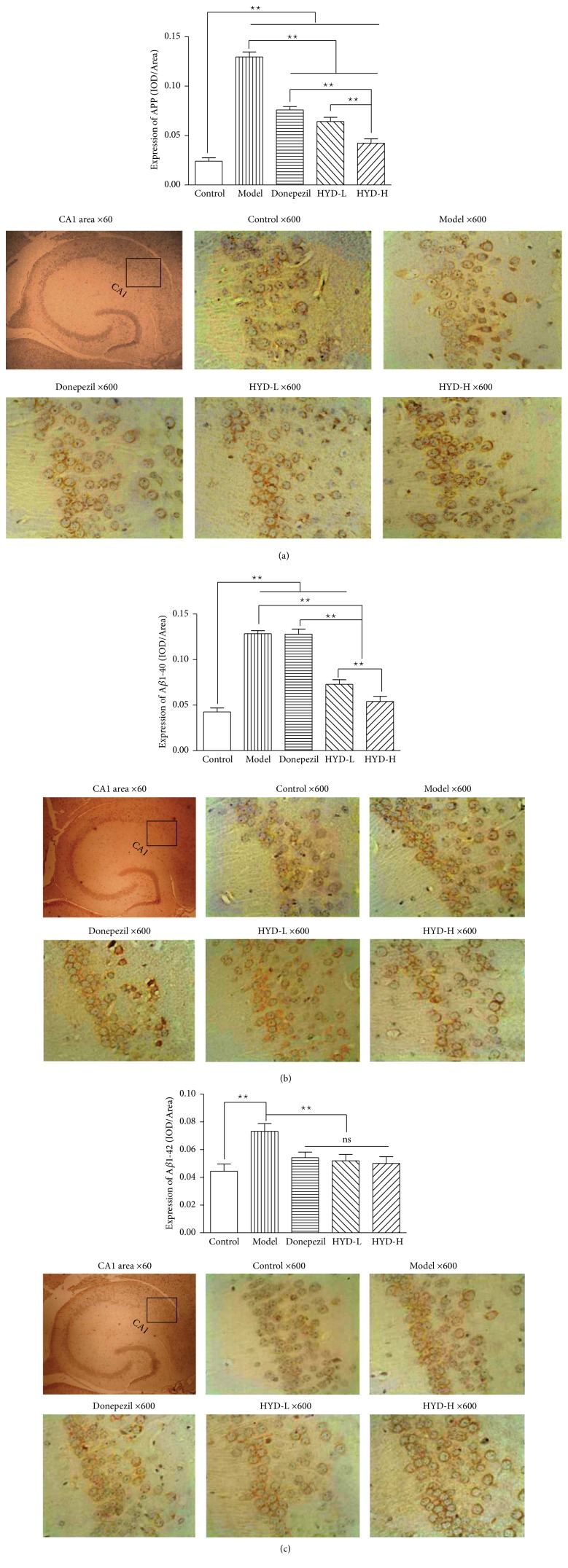
HYD extract regulated protein expression of APP, A*β*1-40, and A*β*1-42 in the hippocampus CA1 area of transgenic APP/PS1 mice (immunohistochemistry, ×60 and ×600). (a), (b), and (c) are the protein expressions of APP, A*β*1-40, and A*β*1-42. ^★★^*P* < 0.01.

Compared with the control group, the expressions of phosphorylated APP and A*β* of model control group mice in hippocampal CA1 area were significantly upregulated (*P* < 0.01); after drug administration, compared with the model group, the expression of phosphorylated APP and A*β*1-42 of Donepezil group, HYD-L group, and HYD-H group and the expression of phosphorylated A*β*1-40 of HYD-L group, and HYD-H group were significantly downregulated (*P* < 0.01); and the expressions of phosphorylated APP and A*β*1-40 of the HYD-H group were significantly lower than the Donepezil group and the HYD-L group (*P* < 0.01) (Figures 4(a), 4(b), and 4(c)).

### 3.4. Effects of HYD Extract on the *γ*-Secretase Activity of Transgenic APP/PS1 Mice

**Figure 5:**
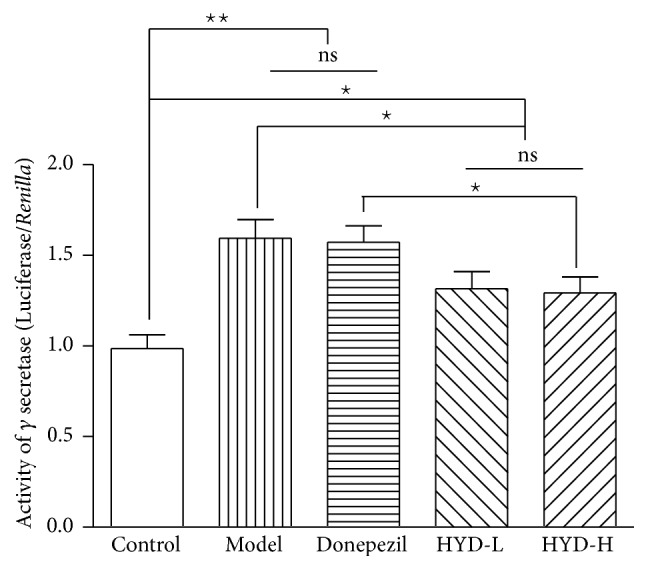
HYD extract regulated activity of *γ*-secretase of transgenic APP/PS1 mice. ^★^*P* < 0.05, ^★★^*P* < 0.01.

Compared with the control group, the *γ*-secretase activity of model group significantly increased (*P* < 0.01); after drug administration, compared with the model group, the *γ*-secretase activity of HYD-L group and HYD-H group decreased (*P* < 0.05); compared with the Donepezil group, the *γ*-secretase activity of HYD-H group decreased (*P* < 0.05) (Figure 5).

### 3.5. Effects of HYD Extract on the Protein Expression of APH-1a, HIF-1*α*, PEN-2, and CREB of Transgenic APP/PS1 Mice

**Figure 6:**
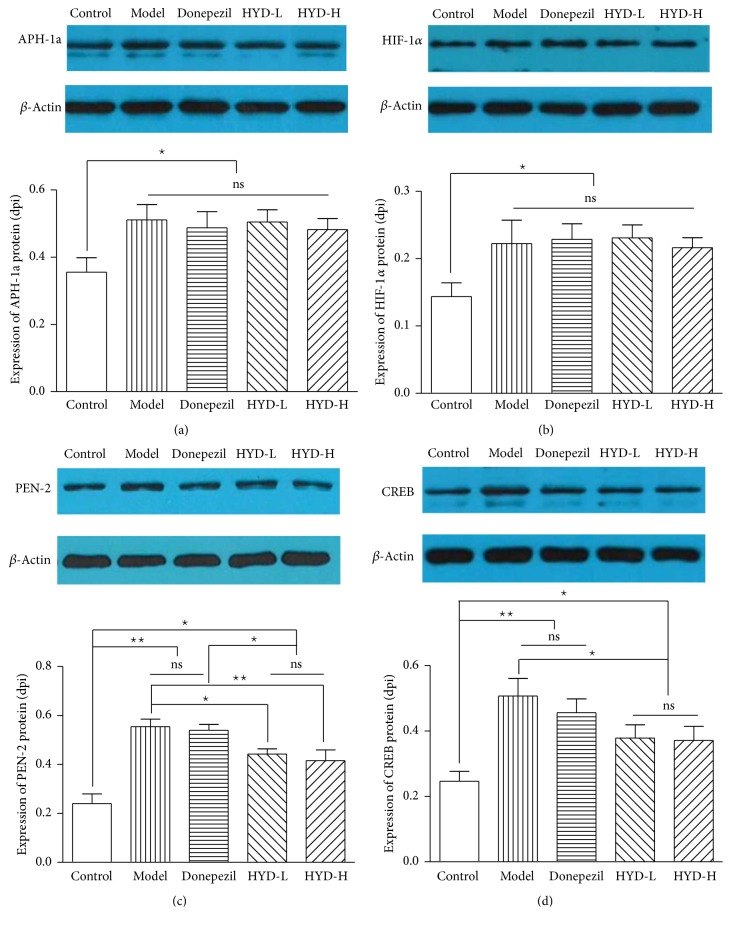
HYD extract regulated protein expression of APH-1a, HIF-1*α*, PEN-2, and CREB of transgenic APP/PS1 mice (Western blotting, APH-1a 1 : 1000, HIF-1*α* 1 : 1000, PEN-2 1 : 200, CREB 1 : 2000, *β*-actin 1 : 5000). (a), (b), (c), and (d) are the protein expressions of APH-1a, HIF-1*α*, PEN-2, and CREB. ^★^*P* < 0.05, ^★★^*P* < 0.01.

Compared with the control group, the protein expressions of APH-1a, HIF-1*α*, PEN-2, and CREB were increased significantly (*P* < 0.05, *P* < 0.01); after drug administration, compared with the model group, the protein expressions of PEN-2 and CREB of HYD-L group and HYD-H group significantly decreased (*P* < 0.05, *P* < 0.01); compared with the Donepezil group, the protein expressions of PEN-2 of HYD-L group and HYD-H group decreased (*P* < 0.05) (Figures 6(a), 6(b), 6(c), and 6(d)).

### 3.6. Effects of HYD Extract on the mRNA Expression of APH-1a, HIF-1*α*, PEN-2, and CREB of Transgenic APP/PS1 Mice

**Figure 7:**
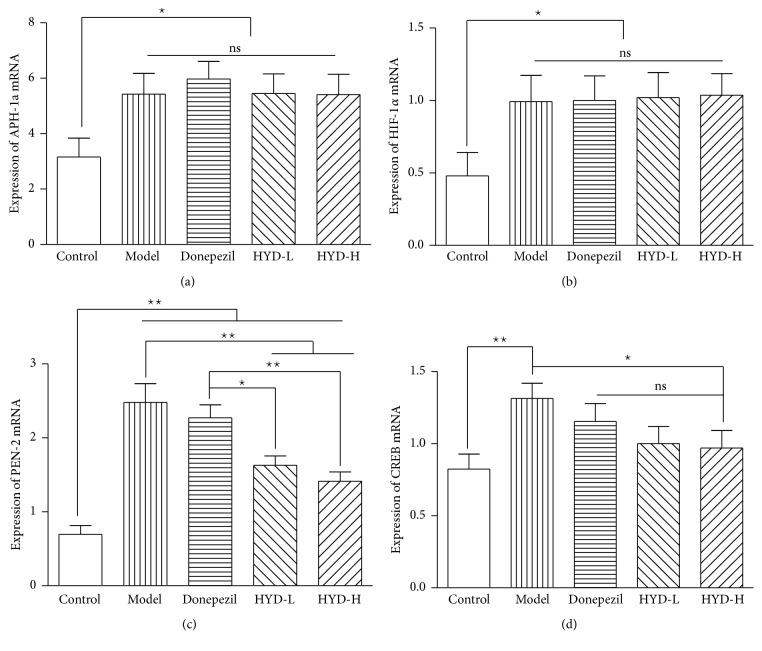
HYD extract regulated mRNA expression of APH-1a, HIF-1*α*, PEN-2, and CREB of transgenic APP/PS1 mice. (a), (b), (c), and (d) are the mRNA expressions of APH-1a, HIF-1*α*, PEN-2, and CREB. ^★^*P* < 0.05, ^★★^*P* < 0.01.

Compared with the control group, the mRNA expression of APH-1a, HIF-1*α*, PEN-2, and CREB of model group increased significantly (*P* < 0.05, *P* < 0.01); after drug administration, compared with the model group, the mRNA expression of PEN-2 of HYD-L group and the mRNA expression of PEN-2 and CREB of HYD-H group significantly decreased (*P* < 0.05, *P* < 0.01); compared with the Donepezil group, the mRNA expression of PEN-2 of HYD-L group and HYD-H group significantly decreased (*P* < 0.05, *P* < 0.01) (Figures 7(a), 7(b), 7(c), and 7(d)).

## 4. Discussion

Alzheimer's disease is a neurodegenerative disease characterized by progressive memory decline and cognitive impairment. The main pathological features of Alzheimer's disease include senile plaques, nerve fiber entanglement, and neuronal loss. The production and deposition of A*β* (especially for A*β*1-42) play an important role in the pathological process. The A*β* deposition in extracellular space directly results in the senile plaques and then further induces tau protein hyperphosphorylation in the cells, forming the neurofibrillary tangles, both of which can cause the corresponding neurotoxic effects, eventually making the structure and function of neurons injured and lost [4, 5]. A*β* is produced through hydrolyzing of amyloid precursor protein (APP) by *β* and *γ* secretase. The *γ*-secretase is the final key of the formation of A*β*, whose activity is closely related to the occurrence of Alzheimer's disease. So the intervention in above pathological process is very important for the prevention and treatment of Alzheimer disease [6].

APP mutation can cause the structural change of APP binding sites with *β*-secretase, which increased *β*-secretase activity, so as to increase the amount of A*β*, and intensifies the formation of senile plaques. PS1 mutation can change *γ*-secretase activity, resulting in the change of the metabolic process of APP, selectively causing A*β*1-42 production and increase. So the transgenic APP/PS1 mice can well simulate human Alzheimer's disease of graduated progress [7]. According to the reports, the 2.5-month-old mice began to show A*β* deposition, around 3-month-old mice began to show cognitive and behavioral changes of early stage, 7-month-old mice showed cognitive impairment, and 9-month-old mice showed dysfunction of spatial memory [8–10]. The pathological mechanism of the model is associated with activation of *γ*-secretase. It is an ideal model for the study of drugs in the regulation of *γ*-secretase pathway and is also a successful transgenic AD model domestically recognized [11].

*γ*-secretase is kind of membrane integrated protease complexes with large molecular weight, which is composed of four subunits: presenilin (PS, catalytic activity center of *γ*-secretase), nicastrin (NCT, the assembly bracket and binding site of substrate of *γ*-secretase), anterior pharynx defective-1 (APH-1, stent partner of NCT), and presenilin enhancer-2 (Pen-2, the assembly molecular wedge of *γ*-secretase complex). The expression changes of the four essential proteins play an important role in *γ*-secretase activity, the production of A*β*, and the onset of AD [12–16]. The research group studied all the four proteins. This thesis mainly summarizes the research results of APH-1 and PEN-2 and their related factors and the research results of NCT and PS were published in Chinese Journal of Pharmacology and Toxicology [17].

APH-1 is one of the essential constituent proteins of *γ*-secretase in mammalian cells, with two highly homologous proteins: APH-la and APH–lb. APH-1a is the main isomer of *γ*-secretase in the process of embryonic development, and lacking APH-la can reduce the production of A*β*. HIF-1 is a transcription regulatory factor of APH-1a, which is composed of subunits HIF-1*α* and HIF-1*β*. The physiological activity of HIF-1 mainly depends on the activity and expression of HIF-1*α* [18, 19]. Studies had found that APH-1a promoter contains the binding sites of HIF-1, and when HIF-1 is activated, the levels of mRNA and protein of APH-1a, *γ*-secretase activity and A*β* production are significantly increased [20].

PEN-2 is widely expressed in specific nerve cells of most brain areas of transgenic APP/PS1 mice, including the olfactory bulb, basal ganglia, striatum, cortex, hippocampus, amygdala, thalamus, hypothalamus, cerebellum, brainstem, and spinal cord. It is not only the main component of *γ*-secretase, but also the modulator of *γ*-secretase activity, playing a key role in the production of A*β*1-42 [21–23]. CREB, a transcription regulatory factor of PEN-2, which can activate some specific genes to produce proteins, strengthens the connection between cells and is closely related to the formation and maintaining of long-term memory [24]. The study found that the promoter of PEN-2 gene contains CREB binding site and can bind to each other specifically, consequently decreasing *γ*-secretase activity and A*β* formation through inhibiting CREB activity and reducing the expression of PEN-2 [25].

The results of this study show that the latency of 9-month-old transgenic APP/PS1 mice in the Morris water maze test was significantly prolonged, and the platform-site crossing times were significantly decreased, which showed that the spatial learning and memory ability were significantly decreased. On the pathological morphology, neurons and Nissl body in hippocampal CA1 area were significantly decreased in quantity and significantly impaired in structure. Meanwhile, *γ*-secretase activity and the expression of APP, A*β*1-40, and A*β*1-42 in hippocampus CA1 area were significantly increased. Furthermore, the APH-la, HIF-1*α*, PEN-2, CREB protein, and their mRNA expression of 9-month-old transgenic APP/PS1 mice were significantly increased. It suggested that HIF-1*α* binding sites of APH-1a promoter and CREB binding sites of PEN-2 promoter in the hippocampal tissue of transgenic APP/PS1 mice were activated, which resulted in a significant increase in APH-1a and PEN-2 expression and *γ*-secretase activity and caused APP dissociation and production of a large number of A*β*. Neurons damage and loss were caused by further accumulation of A*β*, which affected the spatial learning and memory. It was one of the main reasons for the mice to become AD model.

In Traditional Chinese medicine theory, AD is thought as amnesia or dementia which was caused by deficiency of the kidney essence in origin and blood stasis and turbidity in superficiality. So our team proposed that treatment regimens of AD were reinforcing deficiency and supplementing body essence mainly and promoting blood circulation to remove blood stasis and relieving toxicity and transforming turbid secondly. HYD was formulated through above regimen that was composed of Radix polygonum multiflorum preparata,* Panax ginseng*,* Acorus gramineus*,* Coptis chinensis,* and* Ligusticum wallichii*. Early toxicity experiments had confirmed that HYD extract was safe and had no obvious side effects.

After drug administration of six months, HYD extract can significantly shorten the latency and increase the crossing times of transgenic APP/PS1 mice in Morris water maze test; can obviously protect neurons and Nissl body, improve their quantity and structure, and promote the protein synthesis in hippocampus CA1 area; can significantly increase *γ*-secretase activity and the expression of APP, A*β*1-40, and A*β*1-42; can obviously decrease PEN-2, CREB protein, and their mRNA expression and its effect is better than or equivalent to Donepezil. The experiment showed that HYD extract can down-regulate PEN-2 expression and *γ*-secretase activity through inhibiting the CREB binding site activation of PEN-2 gene promoter, intervene in APP metabolic pathways, reduce the production of A*β*1-40 and A*β*1-42, restrain the formation of senile plaques, reduce the loss of neurons, improve memory, and then inhibit AD occurring and developing. So it had a positive meaning for early applying HYD extract to prevent and treat AD. However, HYD extract could not obviously change APH-la, HIF-1*α* protein, and their mRNA expression, which indicated that APH-la and HIF-1*α* are not HYD targets in treating AD.

## 5. Conclusion

HYD extract can obviously improve the spatial learning and memory ability of transgenic APP/PS1 mice. The mechanism was related to its protection of neurons and the structure of Nissl body, the reduction of APP cleavage and A*β* (especially A*β*1-42), and the inhibition of *γ*-secretase activity which results from the inhibition of CREB activity and further downregulated expression of PEN-2.
